# NSAIDs in the Acute Treatment of Migraine: A Review of Clinical and Experimental Data

**DOI:** 10.3390/ph3061966

**Published:** 2010-06-17

**Authors:** Arpad Pardutz, Jean Schoenen

**Affiliations:** 1 Department of Neurology, University of Szeged, Semmelweis u. 6. Szeged, Hungary H-6720, Hungary; E-Mail: apardutz@yahoo.com (A.P.); 2 Headache Research Unit, Department of Neurology & GIGA Neurosciences, Liège University, CHU-Sart Tilman, T4(+1), B36, B-4000 Liège, Belgium

**Keywords:** migraine, NSAIDs, acetaminophen, aspirin, randomized controlled trials, experimental data

## Abstract

Migraine is a common disabling neurological disorder with a serious socio-economical burden. By blocking cyclooxygenase nonsteroidal anti-inflammatory drugs (NSAIDs) decrease the synthesis of prostaglandins, which are involved in the pathophysiology of migraine headaches. Despite the introduction more than a decade ago of a new class of migraine-specific drugs with superior efficacy, the triptans, NSAIDs remain the most commonly used therapies for the migraine attack. This is in part due to their wide availability as over-the-counter drugs and their pharmaco-economic advantages, but also to a favorable efficacy/side effect profile at least in attacks of mild and moderate intensity. We summarize here both the experimental data showing that NSAIDs are able to influence several pathophysiological facets of the migraine headache and the clinical studies providing evidence for the therapeutic efficacy of various subclasses of NSAIDs in migraine therapy. Taken together these data indicate that there are several targets for NSAIDs in migraine pathophysiology and that on the spectrum of clinical potency acetaminophen is at the lower end while ibuprofen is among the most effective drugs. Acetaminophen and aspirin excluded, comparative trials between the other NSAIDs are missing. Since evidence-based criteria are scarce, the selection of an NSAID should take into account proof and degree of efficacy, rapid GI absorption, gastric ulcer risk and previous experience of each individual patient. If selected and prescribed wisely, NSAIDs are precious, safe and cost-efficient drugs for the treatment of migraine attacks.

## 1. Introduction

With a prevalence of 8% in males and 12–15% in females migraine is one of the commonest issues encountered in primary practice [1]. It is characterized by recurrent attacks of pulsatile, unilateral headache often accompanied by nausea and vomiting, photo- and phonophobia. In about 20% of patients the headache is preceeded by an aura consisting of transient neurological symptoms, most frequently a scintillating scotoma. The operational clinical criteria for the diagnosis of migraine with or without aura are defined in International Classification of Headache Disorders (ICHD) (Table 1) [2].

**Table 1:** General diagnostic criteria for Migraine [2].

**Migraine without aura**	
**A.** At least 5 attacks fulfilling criteria B–D	
**B.** Headache attacks lasting 4–72 hours (untreated or unsuccessfully treated)	
**C.** Headache has at least 2 of the following characteristics:	
	-Unilateral location
	-Pulsating quality
	-Moderate or severe pain intensity
	-Aggravation by or causing avoidance of routine physical activity
**D.** During headache at least 1 of the following:	
	-Nausea and/or vomiting
	-Photophobia and phonophobia
**E.** Not attributed to another disorder	
**Migraine with aura**	
**A.** At least 2 attacks fulfilling criteria B–D	
**B.** Aura consisting of at least one of the following, but no motor weakness:	
	-fully reversible visual symptoms including positive features (e.g., flickering lights, spots or lines) and/or negative features ( *i.e.* , loss of vision)
	-fully reversible sensory symptoms including positive features ( *i.e.* , pins and needles) and/or negative features ( *i.e.* , numbness)
	-fully reversible dysphasic speech disturbance
**C.** Art least two of the following:	
	-homonymous visual symmptoms1 and/or unilateral sensory symptoms
	-at least one aura symptom develops gradually over ≥5 minutes and/or different aura symptoms occur in succession over ≥5 minutes
	-each symptom lasts ≥5 and <60 minutes
**D.** Headache fulfilling criteria B–D for migraine without aura begins during the aura or follows aura within 60 minutes	
**E.** Not attributed to another disorder	

Nonsteroidal anti-inflammatory drugs (NSAIDs) are widely available as over-the-counter drugs for pain relief. Most migraine patients have tried at least one of these drugs once in their life to alleviate an attack. NSAIDs are therefore by far the most used class of drugs for the acute treatment of headache in general, and migraine in particular [3,4]. Whereas aspirin (ASA) has been used for the treatment of headaches for many years, the NSAIDs were introduced for this indication more recently. Their usage in migraine is derived from their analgesic properties in other pain disorders and supported by the indirect evidence that prostaglandins are involved in migraine pathophysiology [5]. Many controlled trials have demonstrated the efficacy of the NSAIDs in migraine therapy. We will describe the pharmacology of NSAIDs and its relevance for migraine therapy at the light of experimental data showing that they may play a role in various pathophysiological aspects of migraine, before reviewing the clinical data supporting their use in migraine therapy.

## 2. Pharmacology

**Figure 1:**
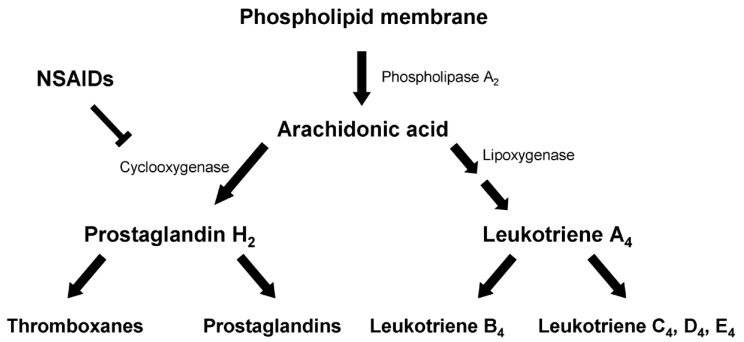
Main biochemical pathways of arachidonic acid. NSAIDs block cyclooxygenase and thus synthesis of prostaglandins from arachidonic acid, but have no effect on lipoxygenase and formation of leukotrienes.

NSAIDs possess anti-inflammatory, analgesic and anti-pyretic proprieties. Their main effect is blockade of the enzyme cyclooxygenase (COX) and hence mitigation of prostaglandin synthesis from arachidonic acid. They have little or no effect on lipoxygenase, leaving leukotriene synthesis intact (Figure 1). Both prostaglandins and leukotrienes are strongly implicated in inflammatory processes [6]. Among the two isoforms of COX, COX-1 is widely distributed and is involved in homeostatic mechanisms, while COX-2 is chiefly expressed in areas of inflammation. The main action of conventional NSAIDs is the non selective inhibition of both isoforms, by contrast with the COX-2 specific NSAIDs which are also effective in migraine. 

Prostaglandins are implicated in sensitization of peripheral nociceptors associated with tissue damage or inflammation. Since NSAIDs inhibit their synthesis, they can be regarded as mild peripheral analgesics and are most effective at sites where inflammation leads to a decreased threshold of polymodal thin fiber nociceptors. In addition, NSAIDs are able to inhibit the synthesis of prostaglandins within the central nervous system, for instance at the level of spinal cord dorsal horns, and to modulate serotonin and catecholamine turnover which contributes to their antinociceptive action.

By blocking platelet cycloxygenase, NSAIDs inhibit the formation of thromboxane A2, a potent aggregating agent. This effect on thrombocytes can be reversible or, as for aspirin, last for the whole life span of the platelet because of acetylation of the enzyme [7]. An exception to this general pharmacological pattern of NSAIDs is acetaminophen, which has only weak anti-inflammatory activity and does not cause clear inhibition of peripheral cyclooxygenase [6].

### 2.1. NSAIDs and Migraine Pathophysiology

#### 2.1.1. Experimental data

##### 2.1.1.1. NSAIDs and peripheral nociceptors in the trigeminovascular system

Activation of the trigeminovascular system (TGV) is a fundamental pain-generating mechanism during the migraine attack. The triggers activating the TGV remain controversial and may be multiple. The activated nociceptors release neuropeptides including calcitonine gene-related peptide (CGRP), sustance P (SP) and neurokinin A [8]. In peripheral blood and saliva, elevated levels of CGRP and SP were observed during migraine attacks in patients and in experimental animal models [9]. The released neuropeptides cause sterile neurogenic inflammation in the dura mater, during the course of which the blood vessels further dilate. Plasma protein extravasation (PPE) occurs, mast cells degranulate, release histamine and polymorphonuclear leukocytes are attracted [10]. These reactions can be observed in experimental models of migraine [11,12]. The released inflammatory substances stimulate trigeminal first-order nociceptors and produce peripheral sensitization [13]. This is thought to correlate during the migraine attack with the throbbing character of the head pain and its aggravation by Valsalva maneuvers, including physical exercise, bending over, coughing or sneezing [14]. 

Many experimental results support the role of the cyclooxygenases in the peripheral activation of the trigeminovascular system. Both COX-1 and COX-2 isoforms are present in the dura mater. COX-1 is found in dural mast cells and small to medium vessels whereas COX-2 can be found in dural macrophages and some CGRP- containing axons [15]. Prostaglandin E2 (PGE2) release from the rat dura mater was observed after chemical or electrical stimulation [8]. ASA treatment can reduce meningeal nociception in rats [16]. Naproxen also attenuated dural nociceptor activation and reduced peripheral nociceptive sensitization [17]. ASA or indometacin attenuate PPE in the dura mater after electrical stimulation of the gasserian ganglion [18] and it was recently shown that the selective COX-2 inhibitor parecoxib is also effective in similar experimental conditions [19]. These results underscore the importance of the cyclooxygenase system in the peripheral arm of the TGV and suggest that NSAIDs can be effective in migraine therapy via an action on these peripheral nociceptors. 

##### 2.1.1.2. NSAIDs and 2nd order trigeminal nociceptors

The persistent activation of second-order nociceptive neurons in trigeminal nucleus caudalis (TNC) leads to central sensitization which during the migraine attack is thought to produce cutaneous allodynia of scalp and face [20,21]. 

CGRP release from the central terminals of the trigeminal sensory neurons is modulated by PGE2 [22]. Both COX-1 and COX-2 are expressed in the spinal cord and the latter is enhanced after inflammatory stimuli [23,24]. Independently from the peripheral blockade of inflammation, ASA was able to attenuate activation of second order trigeminal neurons in the cat after electrical stimulation of the superior sagittal sinus [25]. Parecoxib was found to mitigate c-fos activation in the TNC after electrical stimulation of the Gasserian ganglion [19]. 

After systemic administration of the nitric oxide (NO) donor nitroglycerin (NTG), another animal model of trigeminovascular activation, COX-2 was increased in hypothalamus and lower brain stem [26]. The NTG-induced increase of c-fos expression in rat TNC was attenuated by indomethacin [27] and the NTG-induce increase of neuronal nitric oxide synthase (nNOS) in the same area by ASA [28]. Along the same line, it was shown that NTG- induced overexpressions of nNOS and calmodulin-dependent protein kinase II (CAMK-II) in TNC, thought to mediate central sensitization, were attenuated by COX-2 but not by COX-1 inhibitors [29,30]. These observations are in accordance with the results of Yang *et al*. showing that intrathecal administration of COX inhibitors reduced allodynia induced by trigeminal ganglion compression [31]. 

##### 2.1.1.3. NSAIDs and migraine "generators"

There is undisputable evidence from imaging studies that the migraine attack is associated with activation of brain stem areas including dorsal raphe nucleus (DRN), nucleus raphe magnus (NRM), locus coeruleus (LC), and periaqueductal grey matter (PAG) [32,33,34]. Whether these areas belonging to the pain control system are "generators", as surmised by some, or simply modulators of the head pain, remains controversial.

COX-1 is present in the PAG [35], whereas COX-2 can be found in LC and DRN [36]. In PAG neurons, COX-2 is an important modulator of glycine- and glutamate-induced ion currents suggesting its involvement in pain control [37]. Moreover, COX inhibitors can potentiate opioid inhibition in the PAG [38]. There is also evidence that COX-1 in the PAG can explain a central component in the antinociceptive effects of NSAIDs [39].

##### 2.1.1.4. Cortical spreading depression

Cortical spreading depression (CSD) is important in migraine pathophysiology. It is a well studied slow spreading wave of brief neuron- glial excitation followed by long-lasting neuronal depression [40] which is accompanied by slowly spreading cortical hypoperfusion [41]. There is strong evidence that CSD is the culprit for the migraine aura [42], which includes various transient neurologic symptoms, of which the most common is the visual symptom called scintillating scotoma. In experimental animals, CSD is able to activate the trigeminovascular afferents [43] and to cause PPE in the dura mater [44]. It could thus be able to initiate the above described sensitization process in the trigeminal system. 

The effect of COX inhibition on CSD is controversial. In one experiment ASA failed to modulate CSD in the cat brain [45]. Another work showed that ASA and paracetamol effectively reduced retinal CSD [46]. Pial arteriolar constriction during CSD is mediated by prostanoids in the rabbit [47]. Many studies found a positive correlation between CSD and the expression of COX-2 in the brain [48,49,50]. It is suggested that prostaglandins play an essential role in the downstream events, mostly the vascular changes after CSD which may play a role in migraine and thus offer another target for NSAIDs during the migraine attack.

Taken together the experimental data suggest that both the peripheral and central portions of the TGV are targets for NSAIDs, and can explain their efficacy in experimental models of the migraine headache. In addition, NSAIDs may modulate activity in central pain control systems such as the PAG which may also be relevant for migraine. Their potential effect on CSD and its neurobiological consequences need to be better studied. One has to keep in mind that the precise pathogenesis of migraine itself is not fully understood and that the deductions from experimental studies must be taken with reservations. 

#### 2.1.2. Clinical data

As a matter of fact, early experiments of intravenous prostaglandin infusion [51,52,53,54] provided little support for their involvement in migraine pathophysiology. Infusion of prostacyclin in eight migraine patients induced migraine-like headache in only one case, and mildly worsened an actual attack in two subjects, suggesting that vasodilating prostaglandins were not crucial mediators of vascular headaches [54]. In a more recent study of prostaglandin I2 (epoprostenol), a stable prostacyclin analogue, infusions in healthy volunteers induced headache attributed to vasodilatation while six out of 12 migraine patients without aura developed a typical migraine attack on the day of perfusion associated with superficial temporal artery dilatation and blood flow velocity decrease in middle cerebral artery [55]. Another NSAID effect potentially relevant for migraine prophylaxis is inhibition of platelet aggregation. The beneficial effect of aspirin and NSAIDs in the treatment of migraine attacks most likely results, however, from their combined action on neurogenic inflammation [18], trigeminal nociceptive processing [25] and antinociceptive systems [56] in brainstem and thalamus [57].

### 2.2. Pharmacokinetics

Speed of absorption is a key issue in the pharmacological treatment of migraine attacks, since the holy grail is the fastest possible disappearance of the symptoms. Under normal conditions NSAIDs are quickly absorbed after oral administration with a time to peak plasma concentration (t_max_) of less than 2 hours [6]. Aspirin is absorbed very quickly, with a t_max_ of less than half an hour and is metabolized quickly to salicylic acid [6,58], whereas naproxen has a t_max_ of almost 2 hours. 

Gastric hypomotility during the attack, however, can seriously hamper the rate of drug absorption [59]. To improve absorption, solubility was increased for some NSAIDs (Lys-ASA, diclofenac-K, Naproxen-Na); others were encapsulated as liquid preparations (ibuprofen, diclofenac). An alternative strategy is to combine the NSAID with a prokinetic/antiemetic drug like metoclopramide or domperidone. Although direct comparisons of the absorption of these preparations are rare and quasi exclusively carried out in healthy volunteers, it seems appropriate to choose the most rapidly absorbed formulation of each drug. For migraine attacks with severe nausea or vomiting, rectal or parenteral administration of NSAIDs must be considered [60]

## 3. Results of Randomized Controlled Trials

### 3.1. Placebo Controlled Trials

Many placebo controlled double-blind randomized trials were performed to evaluate the efficacy of NSAIDs or their combination with either metoclopramide or caffeine. The number of patients included in these trials varies greatly. Most studies included both migraineurs with (MA) or without (MO) aura except a few where only MO attacks were treated [61,62,63,64,65]. A crossover design was used in most trials.

**Aspirin** (ASA) has a longstanding history in the treatment of migraine attacks: doses of 500 to 1,000 mg were superior to placebo in 13 trials, one of which evaluated the intravenous route [66]. Despite data showing faster absorption of the ASA+metoclopramide combination [59] one trial failed to demonstrate superiority of ASA+metoclopramide over ASA alone [67]. Highly soluble aspirin salts (900 mg) combined with metoclopramide were superior to placebo [61,65,68,69]. In one trial their effect was comparable to sumatriptan [65]. No significant difference for the primary efficacy parameter—headache relief in the first attack—was detected between a 1,000 mg lysine acetylsalicylic acid—10 mg metoclopramide combination (45%) and 100 mg sumatriptan (56%), but in every other respect and for the second attack sumatriptan was superior. Intravenous aspirin is less effective than subcutaneous sumatriptan, but better tolerated [66]. The ASA+acetaminophen+caffeine combination (600+400+200 mg) had a 26% therapeutic gain over placebo (95% CI 21–31%) when patients with a severe migraine attack were excluded. Compared with placebo, aspirin reduces associated symptoms of nausea, vomiting, photophobia, and phonophobia. 

In a recent systematic Cochrane review of 13 studies, Kirti *et al.* found that a single 1,000-mg dose of aspirin produces headache relief at 2 hours in 52% of attacks, compared to 32% for placebo, while freedom of pain at 2 hours is achieved in 24% of attacks, compared to 11% for placebo. Metoclopramide, when combined with aspirin, significantly reduces nausea and vomiting, but has minimal additional effect on the headache. Aspirin alone is comparable to sumatriptan 50 mg for 2-hour pain-free relief and headache relief, whereas sumatriptan 100 mg is superior to aspirin plus metoclopramide for 2-hour pain-free, but not for headache relief. [70]

**Ibuprofen**, a propionic acid derivative, is a widely used antimigraine drug. Doses of 800 mg to 1,200 mg or 400 mg as an arginine salt were superior to placebo [71,72,73]. Lower doses as a liquigel formulation (200 to 600 mg) were also effective [74], similarly to the 200 and 400 mg conventional formulation [75]. Although significantly better than placebo, 400 mg ibuprofen was less effective than 10 mg rizatriptan [76], but as effective as sumatriptan 50 mg [77].

In low-dose trials, 200 mg tended to be less effective by a small margin. In a trial in children, the effects of ibuprofen and acetaminophen were comparable and they were both better than placebo [78]. In another trial in children, 7.5 mg/kg ibuprofen was effective, but only in boys [79].

**Acetaminophen** alone was efficient at a dose of 1,000 mg [80] while 650 mg was not significantly better than placebo. [81]. Acetaminophen combined to metoclopramide was beneficial compared to placebo [81] as was the acetaminophen + codeine combination [82]. Interestingly, intravenous acetaminophen (1,000 mg) was not found superior to placebo in a German trial [83].

**Tolfenamic acid** was found effective in three trials [84,85,86] and the rapid release form had an efficacy similar to oral sumatriptan [85]. Tolfenamide was as effective as 500 mg ASA and 1 mg ergotamine [84]. A smaller cross-over trial detected a benefit of adding caffeine to tolfenamide [87] but a larger trial showed no difference between tolfenamide+caffeine and tolfenamide alone but detected a slight benefit when tolfenamide was combined with metoclopramide [86].

**Naproxen** was superior to placebo in one trial [88], but in another one it was beneficial only after 2 hours and not for the whole attack [89]. Sodium naproxen, which has better pharmacokinetic proprieties, was superior to placebo in one trial [90]. The combination of naproxen with sumatriptan was clearly superior compared to the single compounds or placebo [91,92,93]. Sumatriptan alone was not superior to naproxen in abolishing pain within 2 hours, but slightly better in pain relief in one of these studies [91].

Enterocoated **diclofenac** 50 mg had marginal efficacy [64] whereas the more rapidly absorbed potassium salt or the sodium salt softgel formulation were superior at doses of 50 mg and 100 mg in several trials [94,95,96,97]. In one placebo-controlled trial, diclofenac K was as effective as caffeine plus ergotamine [96]. There was no increase in efficacy with 100 mg diclofenac compared to 50 mg [94,95] but adding 100 mg caffeine enhanced the efficacy of 100 mg diclofenac softgel but also caused more side effects [97]. Diclofenac sodium was found as effective as oral sumatriptan 100 mg in one study and had less adverse effects [97]. Intramuscular diclofenac [98] was superior to placebo and seems to give better results than the oral formulation, but no direct comparative trials are available [98]. The combination of aceclofenac to almotriptan also showed superiority over the triptan-placebo combination [99].

The effect of **pirprofen** was comparable to an ergotamine combination in one trial and both were superior to placebo [100]. **Flurbiprofen** was superior to placebo in one trial [101]. **Ketoprofen** 75 and 150 mg were compared with 2.5 mg zolmitriptan and placebo in a crossover trial [102]. Zolmitriptan had a slightly higher response rate but all treatments were superior to placebo. Rectal ketoprofen was superior to placebo and to ergotamine in one trial [103]. The COX2 inhibitor **rofecoxib** at doses of 25 mg and 50 mg was superior to placebo in a large parallel group trial [104]; the higher dose was slightly better but at the expense of more side effects. 

**Table 2:** Double-blind, randomized, placebo-controlled trials with NSAIDs in migraine attacks.

Authors	Drugs	N° patients	Results
Tfelt-Hansen *et al.*, 1984 [67]	ASA 650+Met 10 / ASA 650/ Pl	85	Need for rescue medication: ASA+Met (63/92) = ASA (51/86) < Pl (75/95)
			Effect on pain: Met + ASA = ASA > Pl
Henry *et al.*, 1995 [61]	ASA 900+Met 10 / Pl	303	Success rate: ASA + Met (57%) > Pl (19%)
Lange *et al.*, 2000 [68]	ASA 1000 / Pl	343	2 h response: ASA (55%) > Pl (37%) p < 0.001
			2 h pain free: ASA (29%) > Pl (17%) p = 0.007
MacGregor *et al.*, 2002 [69]	ASA 900 / Pl	101	2 h response: ASA (48%) > Pl (19%) p = 0.0005
			2 h pain free: ASA (14%) = Pl (5%)
			3 h pain free: ASA (18%) > Pl (5%) p < 0.05
Tfelt-Hansen *et al.*, 1995 [65]	ASA 900+ Met / Suma 100 / Pl	421	Success rate 1^st^ attack: ASA + Metocl (57%) = Sum (53%) > Pl (24%)
Diener *et al.*, 2004 [76]	ASA 1000 / Suma 50 / Ibu 400/ Pl	312	2 h relief : Ibu (60.2%) = Suma (55.8%) = ASA (52.5%) > Pl
			2 h pain free: Suma (37.1%) = Ibu (33.2%) > ASA (27.1%) > Pl (12.6%)
Diener *et al.*, 1999 [66]	Lys-ASA 1000 iv / Suma 6 s.c.	275	2 h relief: Suma (91%) > Lys-ASA (74%) > Pl (24%)
			2 h pain free: Suma (76.3%) > Lys-ASA (43.7%) > Pl (14.3%)
Havanka-Kanniainen 1989 [ 71]	Ibu 800 (+ 400) / Pl	27	Decrease of attack duration: Ibu (5 h) > Pl (11 h)
			Mild attacks: Ibu (33%) > Pl (7%)
Kloster *et al.*, 1992 [72]	Ibu 1200 / Pl	25	Headache severity: Ibu (1,78) > Pl (2,33)
			Need for rescue medication: Ibu (25.6%) > Pl (57.5%)
Sandrini *et al.*, 1998 [73]	IbuArg 400 / Pl	29	Pain reduction: IbuArg > Pl
Kellstein *et al.*, 2000 [74]	Ibuliq 200, 400, 600 / Pl	735	2 h relief: Ibuliq (64%, 72%, 72%) > Pl (50%)
			2 h pain free: Ibuliq (25%, 28%, 29%) > Pl (13%)
Codispoti *et al.*, 2001 [75]	Ibu 200, 400 / Pl	460	2 h response: Ibu 400 (41%) = Ibu 200 (42%) > Pl (28%)
Misra *et al.*, 2007 [76]	Ibu 400 / Riza 10 / Pl	155	2 h relief: Riza (73%) > Ibu (54%) > Pl (8%)
Hämäläinen *et al.*, 1997 [78]	Ibu 10/kg / Ace 15/kg / Pl (children 4–16 yo)	88	Ibu > Ace > Pl
Lipton *et al.*, 2000 [80]	Ace 1000 / Pl	140	2 h response: Ace (58%) > Pl (39%)
			2 h pain free: Ace (22%) > Pl (11%)
Boureau *et al.*, 1994 [82]	ASA 1000 / Ace 400+Cod 25	198	Success rate: ASA (52%) = Ace + Cod (50%) > Pl (30%)
Leinisch *et al.*, 2005 [83]	Ace 1000 iv / Pl	60	2 h pain free: Ace (10%) = Pl (13%)
			2 h relief: Ace (30%) = Pl (20%)
Hakkarainen *et al.*, 1979 [84]	Tol 200 / Erg 1 / ASA 500 / Pl	20	Duration of attacks: Tol (3,2) = Erg (3,8) = ASA (4,2) > Pl (7,1)
Myllyla *et al.*, 1998 [85]	Tol 200 / Suma 100	141	2 h response: Tol (77%) = Sum (79%) > Pl (29%)
Tokola *et al.*, 1984 [86]	Tol 200 / Tol 200+Met 10/ Tol 200+Caff 100 / Pl	49	Tol + Met > Tol = Tol + Caff > Met = Caf = Pl
Nestvold, *et al.*, 1985 [88]	Napro 750 (+205/500) / Pl	32	Headache relief: Napro > Pl
			Need for rescue medication: Napro (24%) > Pl (46%)
Andersson *et al.*, 1989 [89]	Napro 750 (up to 1250) / Pl	32	2 h relief: Napro > Pl
			Severity for the whole attack: Napro (2,2) = Pl(2,2)
Johnson *et al.*, 1985 [90]	NaproNa 825 (up to 1375 / Pl	61	Relief: NaproNa (3,8) > Pl (5,0)
			Need for rescue medication: NaproNa (44%) < Pl (67%)
Smith *et al.*, 2005 [93]	NaproNa 500 / Suma 50 / NaproNa 500 + Suma 50 / Pl	972	2h relief: NaproNa + Suma (65%) > Suma (49%) = NaproNa (46%) > Pl (27%)
			2h pain free: NaproNa + Suma (34%) > Suma (20%) = NaproNa (18%) > Pl (6%)
Brandes *et al.*, 2007 [91]	NaproNa 500 / Suma 85 / NaproNa 500 + Suma 85 / Pl (2 studies)	1461/1495	2h relief: NaproNa + Suma (65/57%) > Suma (55/50%) > NaproNa (44/43%) > Pl (28/29%)
			2h pain free: NaproNa + Suma (34/30%) > Suma (25/23%) = NaproNa (15/16%) > Pl (9/10%)
Bussone *et al.*, 1999 [94]	DicloK 50, 100 / Suma 100 / Pl	156	2 h relief: DicloK 50 (-17) = Diclo 100 (-18.6) = Suma 100 (-14.5) > Pl
Dahlöf *et al.*, 1993 [95]	DicloK 50, 100 / Pl	73	2 h relief: DicloK 100 = DicloK 50 > Pl
Peroutka *et al.*, 2004 [97]	Diclo 100 / Diclo 100 + Caff 100 / Pl	72	1 h relief: Diclo + Caff (41%) > Diclo (27%) > Pl (14%)
			Need for rescue medication: Diclo + Caff (33%) = Diclo (30%) > Pl (63%)
Massiou *et al.*, 1991 [64]	Diclo 50 (100) / Pl	91	2 h pain free: Diclo (27%) > Pl (19%)
			Need for rescue medication: Diclo (54%) > Pl (66%)
Del Bene *et al.*, 1987 [98]	Diclo 75 im / Pl	32	Response to treatment: Diclo > Pl
Kinnunen *et al.*, 1988 [100]	Pirpro 200 (500) / Erg 2 (5) / Pl	55	Pain relief: Pirpro = Erg > Pl
			Need for rescue medication: Pirpro (18/58) = Erg (18/59) < Pl (32/60)
Awidi *et al.*, 1982 [101]	Flurbi 100 (300) / Pl	19	Relief score: Flurbi (3,2) > Pl (0,7)
Dib *et al.*, 2002 [102]	Keto 75, 150 / Zolmi 2, 5 / Pl	235	2 h relief: Zolmi (67%) = Keto 150(62%) = Keto 75 (63%) > Pl
Silberstein *et al.*, 2004 [104]	Rof 25, 50 / Pl	557	2 h relief: Rof 50 (57%) = Rof 25 (54%) > Pl (34%)

*Abbreviations:* Ace: acetaminophen; ASA: acetylsalicylic acid-aspirin; Caff: caffeine; Diclo: diclofenac; DicloK: diclofenac-potassium; Erg: ergotamine; Ibu: ibuprofen; IbuArg: ibuprofen arginate; Ibuliq: ibuprofen liquigel; Indo: indomethacin; Flurbi: flurbiprofen; Keto: ketoprofen; Met: metoclopramide; Napro: naproxen; NaproNa: naproxen-sodium; Pirpro: pirprofen; Pl: placebo; Rof: rofecoxib; Suma: sumatriptan; Tol: tolfenamic acid; Zolmi: zolmitriptan.

### 3.2. Comparative Trials

Aspirin was inferior to ergotamine and to a dextropopoxyphene compound for the treatrment of migraine attacks [105,106]. The ASA 900 mg + metoclopramide 10 mg combination was as effective as 2.5 mg zolmitriptan regarding 2-hour pain relief, but inferior to the triptan for other outcome measures such as 2-hour pain-free rates [107].

Ibuprofen proved superior to acetaminophen in one trial [108] and intramuscular diclofenac was superior to intramuscular acetaminophen in another one [109]. Mefenamic acid was not superior to acetaminophen when each was combined with metoclopramide [110]. 

Tolfenamic acid at 200 and 400 mg was superior to acetaminophen in a medium-sized cross-over trial with 58 patients [63] without a significant difference between the two dosages. 

One trial showed some superiority of naproxen sodium over an ergotamine combination [111] but in other studies equal effects were found for naproxen sodium and ergotamine [112] or ergotamine + caffeine [113].

Intramuscular ketorolac 30 mg had less efficacy than 75 mg meperidine [114], but a dose of 60 mg was as effective as 75 mg meperidine (plus 25 mg promethazine) [115] or 100 mg meperidine (plus 50 mg hydroxyzine) or 25 mg chlorpromazine iv [116] in rather small randomized trials performed in emergency departments (n = 30 to 47 patients included). Ketorolac 30 mg iv (n = 64) was inferior to 10 mg iv prochlorperazine [117], but more effective than 20 mg nasal sumatriptan (n = 29) [118]. Ketoprofen 100 mg im was superior to 500 mg im acetaminophen [103]. 

The oral combination of indomethacin, caffeine and prochlorperazine had similar efficacy compared to oral sumatriptan [119], whereas in an open randomized study the rectal combination of these drugs was superior to rectal sumatriptan [120]. 

**Table 3:** Comparative trials with NSAIDs in migraine attacks.

Trial	Drug	N° patients	Results:
Geraud *et al.*, 2002 [107]	ASA 900+Met 10 / Zolmi 2,5	666	2 h relief: ASA + Met (32.9%) = Zol (33.4%)
			2 h pain free: Zolmi (10.7%) > ASA + Met (5.3%)
Tfelt-Hansen *et al.*, 1995 [65]	ASA 900+ Met / Suma 100 / Pl	421	Success rate 1^st^ attack: ASA + Metocl (57%) = Sum (53%) > Pl (24%)
Diener *et al.*, 2004 [76]	ASA 1000 / Suma 50 / Ibu 400/ Pl	312	2 h relief : Ibu (60.2%) = Suma (55.8%) = ASA (52.5%) > Pl
			2 h pain free: Suma (37.1%) = Ibu (33.2%) > ASA (27.1%) > Pl (12.6%)
Diener *et al.*, 1999 [66]	Lys-ASA 1000 iv / Suma 6 s.c.	275	2 h relief: Suma (91%) > Lys-ASA (74%) > Pl (24%)
			2 h pain free: Suma (76.3%) > Lys-ASA (43.7%) > Pl (14.3%)
Misra *et al.*, 2007 [76]	Ibu 400 / Riza 10 / Pl	155	2 h relief: Riza (73%) > Ibu (54%) > Pl (8%)
Hämäläinen *et al.*, 1997 [78]	Ibu 10/kg / Ace 15/kg / Pl (children 4–16 yo)	88	Ibu > Ace > Pl
Karachalios *et al.*, 1992 [109]	Diclo 75 im / Ace im.	86	30 min pain free: Dicl (88%) > Ace (17.5%)
Bussone *et al.*, 1999 [94]	DicloK 50, 100 / Suma 100 / Pl	156	2 h relief: DicloK 50 (-17) = Diclo 100 (-18.6) = Suma 100 (-14.5) > Pl
Schoenen *et al.*, 2008 [99]	Aceclo 100 + Almo 12.5 / Almo 12.5 + Pl	112	2 h relief: Aceclo + Almo (69%) > Almo + Pl (57.9%)
			2 h pain free: Aceclo + Almo (40.7%) > Almo + Pl (29.1%)
Hakkarainen *et al.*, 1979 [84]	Tol 200 / Erg 1 / ASA 500 / Pl	20	Duration of attacks: Tol (3,2) = Erg (3,8) = ASA (4,2) > Pl (7,1)
Myllyla *et al.*, 1998 [85]	Tol 200 / Suma 100	141	2 h response: Tol (77%) = Sum (79%) > Pl (29%)
Larsen *et al.*, 1990 [63]	Tol 200,400 / Ace 500,1000	83	2 h effect on pain: Tol > Ace
Pradalier *et al.*, 1985 [111]	Napro 825 / Erg 2+Caff 91.5+Cyclizine 50	114	If taken within 2 h of onset: Napro > Erg + Caff + C
Treves *et al.*, 1992 [112]	Napro / Erg	42	Overall patients' satisfaction: Napro > Erg
			Duration and severity reduction: Napro = Erg
Sargent *et al.*, 1988 [113]	Napro 825 / Erg 1+Caff 100		Attack abortion: Napro = Erg + Caff > Pl
			Nausea reduction: Napro > Erg + Caff
Smith *et al.*, 2005 [93]	NaproNa 500 / Suma 50 / NaproNa 500+Suma 50 / Pl	972	2 h relief: NaproNa + Suma (65%) > Suma (49%) = NaproNa (46%) > Pl (27%)
			2 h pain free: NaproNa + Suma (34%) > Suma (20%) = NaproNa (18%) > Pl (6%)
Brandes *et al.*, 2007 [91]	NaproNa 500 / Suma 85 / NaproNa 500+Suma 85 / Pl (2 studies)	1461/1495	2 h relief: NaproNa + Suma (65/57%) > Suma (55/50%) > NaproNa (44/43%) > Pl (28/29%)
			2 h pain free: NaproNa + Suma (34/30%) > Suma (25/23%) = NaproNa (15/16%) > Pl (9/10%)
Kinnunen *et al.*, 1988 [100]	Pirpro 200 (500) / Erg 2 (5) / Pl	55	Pain relief: Pirpro = Erg > Pl
			Need for rescue medication: Pirpro(18/58) = Erg (18/59) < Pl (32/60)
Karabetsos *et al.*, 1997 [103]	Keto 100 im / Ace 500 im	64	40 min relief: Keto (82.5%) > ace (17.5%)
Dib *et al.*, 2002 [102]	Keto 75, 150 / Zolmi 2,5 / Pl	235	2 h relief: Zolmi (67%) = Keto 150 (62%) = Keto 75 (63%) > Pl
Larkin *et al.*, 1992 [114]	Ketorolac 30 im / Meperidine 75 im	31	1 h relief: Meperidine > Ketorolac
Davis *et al.*, 1995 [115]	Ketorolac 60 im / Meperidine 75+ promethazine 25 im	42	30 min response: Meperidine + promethazine (68%) = Ketorolac (55%)
Shrestha *et al.*, 1996 [116]	Ketorolac 60 im / Chlorpromazine 25 iv	47	30 min-2h relief: Ketorolac = Chlorpromazine
Seim *et al.*, 1998 [117]	Ketorolac 30 iv / Prochlorperazine 10 iv	64	1 h relief: Prochlorperazine > Ketorolac
Meredith *et al.*, 2003 [118]	Ketorolac 30 iv / Suma 20 nasal	29	1 h relief: Ketorolac > Suma
Sandrini *et al.*, 2007 [119]	Indo 25+Prochlor 2+Caff 75 / Suma 50	297	2 h relief: I + P + C (57%) = Suma (57%)
			2 h pain free: I + P + C (32%) = Suma (36%)
Di Monda *et al.*, 2003 [120]	Indo 25 + Prochlor 2 + Caff 75 rectal / Suma 25 rectal	88	2 h relief: I + P + C (67%) = Suma (63%)
			2 h pain free: I + P + C (47%) > Sum (35%)

*Abbreviations:* Ace: acetaminophen; Aceclo: aceclofenac; Almo: almotriptan; ASA: acetylsalicylic acid-aspirin; Caff: caffeine; Diclo: diclofenac; DicloK: diclofenac-potassium; Erg: ergotamine; Ibu: ibuprofen; Indo: indomethacin; Keto: ketoprofen; Met: metoclopramide; Napro: naproxen; NaproNa: naproxen-sodium; Pirpro: pirprofen; Suma: sumatriptan; Riza: rizatriptan; Tol: tolfenamic acid; Zolmi: zolmitriptan.

### 3.3. Summary of Efficacy Data

Taken together, there is level A evidence that overall NSAIDs are effective for the treatment of migraine attacks. Although there are few scientific data on dose-response relationships, higher doses seem to be more effective.

Due to the low number of comparative trials and the small sample size in many trials, there is little scientific reason to prefer one NSAID over the other, but ASA and NSAIDs like ibuprofen, were in all available trials superior to acetaminophen. The selection of an NSAID should take into account the side effect profile in addition to efficacy. It is not known whether it is worth trying another NSAID after failure of one. 

Except for the combined indomethacin preparation mentioned above, there are unfortunately no trials with rectal preparations of NSAIDs which might be more adequate and effective than tablets for severe attacks, especially if the patient has nausea or vomiting. 

Parenteral administration of NSAIDs is an effective treatment for migraine in the emergency room and in disabling attacks resisting to oral therapies. 

### 3.4. Adverse Effects

NSAID-induced side effects were minor in all trials and GI tract-related, like epigastric pain. In a large survey by Lanas *et al.* [121] the age-adjusted relative risk of presenting upper GI bleeding was on average 5.3 for NSAIDS; it was lowest for aclofenac (1.4), ibuprofen (2.5), indomethacin (3.3) and naproxen (4). It was on average 1.5 for selective COX-2 inhibitors, a difference with conventional NSAIDs also confirmed in another study [122]. 

As expected from earlier studies [87,123], tolerability of NSAIDs was superior to that of ergotamine in several trials [62,84,100,111,112,113], while efficacy was similar. Ergotamine caused more nausea and vomiting, although this was statistically significant only in two trials [84,111]. When triptans and NSAIDS were compared directly, adverse effects tended overall to be more prevalent with triptans [66,67].

The potential cardiovascular risk associated with the use of COX-2 inhibitors remains controversial. In a large trial a higher cardiovascular risk was associated with the use of rofecoxib compared to naproxen [124] but pooled analyses showed no significant difference in cardiovascular events [125]. More recently the non-selective COX inhibitors were also suspected to increase cardiovascular risk, but clear evidence for this is still missing [126]. Meanwhile NSAIDs should be used with caution in patients with a high cardiovascular risk profile.

A well known complication of frequent intake of symptomatic drugs for migraine is headache chronification leading to so-called medication overuse (MOH). Various studies have shown that NSAIDs have a lower propensity to induce MOH compared to combination analgesics and triptans [127,128]. 

## 4. Conclusions

Aspirin, acetaminophen and other NSAIDs are the most frequently used drugs for the treatment of any headache, including migraine, and many patients use them as over-the-counter drugs. There is evidence from RCTs that acetaminophen is slightly less efficacious for migraine attacks than other NSAIDs. Before considering that NSAIDs are not effective, one has to verify that the dose was high enough and that the drug was taken as soon as the headache appeared. Low bioavailability due to gastroparesis might be a cause for treatment failure; it can be optimized by adding a prokinetic drug like metoclopramide or domperidone to the NSAID, or by switching to rectal or parenteral administration. Intramuscular injections are particularly useful in the emergency situation. 

Although there is no evidence that one NSAID is more effective than another, with the notable exception of acetaminophen, one may recommend to select a drug with a rapid GI absorption and with the most favorable efficacy/side effect profile. Based on these criteria, ace-/diclofenac, ibuprofen, indomethacin and naproxen seem to be good choices. COX-2 inhibitors cause less GI toxicity than nonselective drugs, but their therapeutic efficacy is not superior and some of them may increase vascular risk. Their usage is thus justified only in migraineurs who cannot tolerate non-selective NSAIDs. 

If NSAIDs are not sufficiently effective, triptans are next to recommend. Although in RCTs oral triptans were not uniformally superior to NSAIDs, they are clearly more efficient in clinical practice for the more severe attacks. If an oral triptan is not efficient enough, it can be combined with an NSAID to improve early outcome and to decrease headache recurrence at longer delays. Finally, NSAIDs can be used, with parsimony, as acute treatments during detoxification in migraineurs overusing triptans, combination analgesics or ergotamine [129,130]. 

Despite advances in migraine-specific drugs like triptans and the up-coming CGRP antagonists, NSAIDs remain first choice drugs for the treatment of migraine attacks, especially if they are mild or moderate in intensity.
